# Adolescents’ Identity Formation: Linking the Narrative and the Dual-Cycle Approach

**DOI:** 10.1007/s10964-019-01096-x

**Published:** 2019-08-12

**Authors:** Lotte van Doeselaar, Kate C. McLean, Wim Meeus, Jaap J. A. Denissen, Theo A. Klimstra

**Affiliations:** 1 Department of Developmental Psychology, Tilburg University, Tilburg, The Netherlands; 2 Department of Psychology, Western Washington University, Bellingham, WA USA; 3 Research Centre Adolescent Development, Utrecht University, Utrecht, The Netherlands

**Keywords:** Identity formation, Narrative identity, Autobiographical reasoning, Agency, Commitment, Exploration

## Abstract

The narrative and dual-cycle approach conceptualize and operationalize adolescents’ identity formation in different ways. While the narrative approach focuses on the construction of an autobiographical life story, the dual-cycle approach focuses on the formation of identity commitments. Although these approaches have different emphases, they are conceptually complementary. Yet, their empirical links and distinctions have only scarcely been investigated. Empirical knowledge on these links in adolescence and across time has been especially lacking. In the present research, it was therefore examined whether key characteristics of adolescents’ narration (autobiographical reasoning and agency) were concurrently and prospectively related to engagement in the dual-cycle processes of commitment making, identification with commitment, exploration in breadth, exploration in depth, and ruminative exploration. The findings from a cross-sectional sample of 1,580 Dutch adolescents (*M*_age_ = 14.7 years, 56% female) demonstrated that autobiographical reasoning was significantly positively associated with the commitment and more adaptive exploration processes (i.e., in breadth and in depth). In addition, agency was significantly positively associated with the commitment processes and exploration in depth. Yet, these associations between the narrative characteristics and dual-cycle processes were only weak. Subsequently, the findings from a two-year longitudinal subsample (*n* = 242, *M*_age_ = 14.7 years, 62% female) indicated that on average commitment strength remained stable but exploration increased across middle adolescence. A stronger increase in identification with commitment and adaptive exploration (i.e., in breadth and in depth) was predicted by a higher degree of agency in adolescents’ narratives. Overall, these findings indicate that both approaches to identity formation are associated, but the small size of these associations suggests that they predominantly capture unique aspects of identity formation. Both approaches could thus complement and inform each other.

## Introduction

The construction of a personal identity, the primary task of adolescence (Erikson 1950), is captured in quite distinct ways by two contemporary developmental theories and related methodologies. The narrative approach focuses on the formation of a coherent life story (McAdams 2001). The dual-cycle approach focuses on the formation and evaluation of identity commitments (Luyckx et al. 2006). These two approaches have different emphases, particularly in methodology, but are conceptually complementary (McLean and Pasupathi 2012). For example, they might concurrently capture similar processes of identity formation but with different concepts and they might predict developments in each other over time. So far, empirical studies have only captured a fraction of what an integration of these approaches could offer. Empirical studies on links and distinctions between both approaches in adolescence and between the approaches over time are especially lacking. In the present research, it was therefore examined whether key dimensions of both approaches were concurrently associated in a large cross-sectional Dutch adolescent sample (Study 1). In addition, it was examined whether key dimensions of the narrative approach were predictive of adolescents’ developments in the dimensions of the dual-cycle approach over time in a longitudinal subsample (Study 2).

### Erikson’s Views on Identity Formation

The narrative and the dual-cycle approach to the study of identity formation both originated from the writings of Erik H. Erikson, who derived his ideas from clinical observations and his work on psychobiographies. According to Erikson (1968), identity formation is a lifelong process, which becomes increasingly salient during adolescence. This increased salience is triggered by physical changes during puberty, cognitive development, and societal opportunities and expectations. An explicit current example of such a societal expectation is that in the Dutch educational system adolescents have to make choices in their secondary school curriculum at the end of the second or third year (at about age 14–15). By making these choices, adolescents might already rule out specific tertiary educational tracks (see Klimstra et al. 2012). Triggered by such factors, adolescents become increasingly engaged in (re-)defining themselves in various domains (e.g., occupation, sexuality, ethnicity). Erikson stated that this process takes place within socio-cultural contexts with other people supporting, testing, and (not) recognizing adolescents’ identities, with a variety of identity options, but also with constraints offered. By constructing their own personal identity, adolescents can obtain a sense of “progressive continuity between that which [they have] come to be during the long years of childhood and that which [they promise] to become in the anticipated future” (Erikson 1968, p. 87). This sense of personal continuity is one of the key aspects of Erikson’s conceptualization of an adaptive identity, which is still present in the narrative (McAdams 2001) and dual-cycle approaches (Van Doeselaar et al. 2018). Moreover, both approaches also still recognize the importance of social (e.g., McLean and Jennings 2012; Van Doeselaar et al. 2016) and cultural (e.g., McLean and Syed 2015; Negru‐Subtirica et al. 2017) contexts for identity formation. Yet, in both approaches distinct, but complementary, theoretical ideas on the conceptualization of an (adaptive) personal identity and how a personal identity is constructed and maintained have been added to those of Erikson.

### Narrative Identity

According to McAdams’ life story model of identity, a coherent personal identity is an internalized life story that answers the question who one is and integrates one’s past, present, and future (McAdams 2001). By creating this integrated personal life story individuals can obtain a feeling of personal continuity (McAdams 2001). A coherent life story, or narrative identity, is deemed to be constructed through the process of narrating momentous events of one’s life. Therefore, the characteristics of individuals’ autobiographical stories, typically those stories that are central in one’s life (critical event narratives; Habermas and Reese 2015), are studied to investigate identity formation in the narrative identity approach. The narrative identity approach has a relatively broad view on what an adaptive identity entails, compared to the dual-cycle approach. Not only narrative characteristics that contribute to feelings of personal continuity are theorized to be adaptive (e.g., presence of self-event connections; Pasupathi et al. 2007), but the same is true for other characteristics that are linked with higher well-being (e.g., expressions of agency; Adler 2012).

#### Self-event connections

Self-event connections are a common assessment of autobiographical reasoning (Pasupathi et al. 2007). Autobiographical reasoning entails linking personally important experiences to each other and to aspects of the self and is necessary to create a coherent and integrated life story (Habermas and Reese 2015). In childhood, individuals are generally already able to narrate coherently about a single event (Habermas and Reese 2015). Yet, it is in adolescence that the ability to narrate a coherent life story, in which personally meaningful events and the self are integrated, emerges and continues to develop (Köber et al. 2015). This growth in autobiographical reasoning abilities likely not only occurs because of an emerging urge to define the self (McAdams and McLean 2013), but also because necessary cognitive abilities emerge in adolescence that allow for this kind of integrative work (Habermas and Reese 2015).

In a self-event connection an individual explicitly links an experienced event to one’s sense of self (Pasupathi et al. 2007). An example of this would be stating that an event (e.g., a successful dating experience) caused an enduring change in the self (e.g., becoming less shy). Self-event connections can promote a feeling of personal continuity, because they can both explain personal change over time (e.g., how one became less shy) and mark stability in the self (e.g., not approaching others at various occasions illustrates that one has always been shy; Habermas and Reese 2015). Although self-event connections could entail negative views on the self (McLean and Pasupathi 2011), they are theorized to be highly important for the development of a coherent narrative identity (Pasupathi et al. 2007) and are in that sense considered adaptive.

#### Agency

Apart from the importance of autobiographical reasoning, motivational themes captured in personal narratives have shown to be a key marker of narratives (McLean et al. 2019), and their role in psychological health (Adler 2012). One of these motivational themes that has been consistently linked to well-being is agency (Adler et al. 2016). Agency refers to strivings of individuals to protect and assert themselves, to be autonomous, and to be in control (Bakan 1966). In narratives that are considered to reflect a high degree of agency, individuals describe themselves as able to influence the course of their life (Adler 2012). Agency is likely linked to well-being because describing oneself as agentic satisfies individuals’ needs to feel competent in effecting their lives and to behave in concordance with their sense of self (Adler 2012; Deci and Ryan 2000). Narrating about oneself in an agentic manner might therefore be important to create an adaptive narrative identity. However, the importance of agency might go beyond narrative identity, as agency has been theorized to be linked with the ability to construct satisfying identity commitments (Côté 1997). Consequently, the present research focused on how agency, as well as self-event connections, relate to processes of commitment and exploration, as captured in the dual-cycle model of identity formation.

### Dual-Cycle Model of Identity Formation

Dual-cycle models offer a different perspective on adolescents’ identity formation and view identity not as a life story, but as a set of identity commitments. Identity formation is conceptualized as a dual-cycle process of forming and evaluating identity commitments in various relevant domains, such as occupational aspirations (Crocetti et al. 2008; Luyckx et al. 2006). Strong identity commitments are assumed to provide a sense of personal continuity as they provide a sense of direction and certainty regarding the future (Van Doeselaar et al. 2018). Dual-cycle models provide a more detailed approach to Marcia’s (1966) original identity status framework. This framework provided an empirical definition of identity based on Erikson’s writings. Although the original dimensions of Marcia’s (1966) identity status approach were often assessed by coding interviews, a method that is closer to the methodology of the narrative approach (see Carlsson et al. 2015), the dual-cycle dimensions are currently mostly assessed with self-report questionnaires. The present research focused on the five processes and two cycles articulated by Luyckx et al. (2008). The first cycle focuses on the formation of identity commitments, which reflect choices made in identity relevant domains. This cycle encompasses the processes of *exploration in breadth* and *commitment making*. That is, various options to which one could commit are explored and if a satisfying option is found a commitment is made. The second cycle focuses on the evaluation of commitments and encompasses *exploration in depth* and *identification with commitment*. Current commitments are explored by talking with others and reflecting on them. If the outcome of this evaluation is positive, people will increasingly identify with this commitment and integrate it in the self. If the outcome is unsatisfying, however, the first cycle of broader exploration of alternatives might be re-activated.

The processes of exploration in breadth and in depth represent more adaptive processes of exploration, but in both cycles adolescents might also explore in a more maladaptive, ruminative way if they get stuck in a cycle of repetitively thinking over identity options in a passive manner. This is referred to as *ruminative exploration* and is associated with internalizing problems (Luyckx et al. 2008).

#### Relation of dual-cycle processes to self-event connections

As stated above, the process of making self-event connections is thought to be a form of autobiographical reasoning by which individuals construct their narrative identity and self-concept (Pasupathi et al. 2007). As such, autobiographical reasoning might strengthen adolescents’ commitments (McLean and Pasupathi 2012). By reflecting on the self and constructing a coherent personal life story, adolescents might become better able to make identity choices that fit them. That is, they might use self-ascribed characteristics from their life story (e.g., this event has revealed to me that I am a caring person) in constructing new or strengthening current commitments (e.g., aspiring to a career in nursing). Therefore, adolescents who make self-event connections might not only have stronger commitments concurrently, but might also show an increase in commitment strength over time.

Moreover, the autobiographical reasoning process of making self-event connections could be viewed as an expression of the identity exploration processes (McLean and Pasupathi 2012). Adolescents who engage in identity exploration might reflect more on their self-views than adolescents who are not engaged in identity exploration. For example, they might explore their self-concept and identity by reflecting on and learning from experienced events. Accordingly, adolescents who engage more in identity exploration might make more self-event connections concurrently.

Previous studies have examined the link between autobiographical reasoning and commitment and exploration, but have focused on young adulthood, not adolescence (Carlsson et al. 2015; Carlsson et al. 2016; Glavan et al. 2019; McLean and Pratt 2006; McLean et al. 2016; McLean et al. 2014; Merrill et al. 2016). Overall, these previous findings indicated small associations between engagement in autobiographical reasoning and the commitment and exploration processes in young adulthood. The present research builds on this previous work by examining the concurrent links between autobiographical reasoning and commitment and exploration in adolescence to determine whether these associations are equally small, smaller, or stronger in adolescence than in young adulthood. Moreover, to the best of our knowledge, the present research was the first to examine the hypothesized prospective effects of autobiographical reasoning on the development of commitment strength.

#### Relation of dual-cycle processes to agency

Because the construction of identity commitments is a complex and difficult developmental task, which can make adolescents feel uncertain of who they are, viewing oneself as agentic may be helpful for adolescents to manage this task (Koepke and Denissen 2012; Schwartz et al. 2005). For example, viewing oneself as agentic might stimulate adolescents to explore their options and to commit to the option that they view as fitting them best. Moreover, narrating a critical event in a way that expresses personal agency could be especially related to further adaptive identity development because individuals often rely on memories of past events to guide their actions (Philippe et al. 2011). Adolescents who describe themselves as taking control in events that have been critical for their identity might rely on these narratives in new situations that are important for their identity formation. For instance, a narrative about how an adolescent took the initiative to end an unhealthy friendship might stimulate this adolescent to explore and commit to identity options that more clearly align with relational values. Thus, adolescents who express more agency in critical event narratives than others might be more strongly committed and engage in more adaptive exploration, concurrently and over time.

There are few studies examining narrative agency and commitment and exploration of which we are aware, and none in adolescence. Existing research suggests that agency was more likely to increase in the life stories of young adults who were characterized by strong commitments and exploration (i.e., achieved identity) across time, compared to those who were committed but lacked exploration (i.e., foreclosed identity) or lacked both commitment and exploration (i.e., diffused identity; Carlsson et al. 2015, 2016). Overall, these limited data from young adults indicate that expressing agency in autobiographical narratives might be positively related to adaptive engagement in the formation and evaluation of commitments.

## Current Study

The aim in the present research was to examine links between key dimensions of the narrative and the dual-cycle approach to the study of identity formation in adolescence. This research consisted of two stages. First, concurrent associations were examined in a large cross-sectional adolescent sample (Study 1). Next, predictive effects of narrative characteristics on the dual-cycle processes over time were examined in a subsample of adolescents (also included in Study 1) who participated in a subsequent two-year longitudinal study (Study 2). In both studies, adolescents who made a self-event connection in a critical event narrative were compared to those who did not make a self-event connection. In addition, adolescents were compared based on the degree of agency expressed in their critical event narrative. Because making self-event connections is viewed as a way of forming one’s identity, it was expected that making a self-event connection would be associated with stronger commitments (i.e., commitment making and identification with commitment) concurrently and a more positive increase in commitment strength over time. Additionally, because making self-event connections could be an expression of identity exploration, agency was expected to be positively associated with engagement in identity exploration (in breadth, in depth, and ruminative) concurrently. A predictive effect of making a self-event connection on changes in exploration was examined in an exploratory manner. Moreover, viewing oneself as agentic and being able to rely on critical event narratives in which the self was agentic was thought to stimulate adaptive formation and evaluation of commitments. Therefore, it was expected that agency would be associated with stronger commitments and more adaptive exploration (i.e., more exploration in breadth and in depth, and less ruminative exploration) concurrently and with more positive increases in commitment strength and engagement in adaptive exploration over time (e.g., relatively more positive changes in exploration in breadth and in depth, and less positive changes in ruminative exploration).

## Study 1

### Method

#### Participants and procedure

Data for Study 1 were drawn from the first wave of Project-Me, which took place in 2015 and 2016. Participants were recruited from the second and third year of various secondary schools in the Netherlands. Secondary education in the Netherlands is primarily captured by three main levels: pre-vocational education, higher general secondary education, and preparatory scientific education, hereafter referred to as the relatively lower, medium, and higher educational levels, respectively. Participants of classes from all three educational levels were included in Project-Me. Two weeks before participation, parents received information on the study with information on how to opt their child out of the study. Trained research assistants visited adolescents’ classes to introduce the study, after which adolescents engaged in a process of consent if they wanted to participate. Of all possible adolescents in the targeted classes, 91% participated in the study. Adolescents who did not participate were not present, had no parental consent, or decided not to participate themselves.

Participants completed an online questionnaire during one class hour (i.e., 45 or 50 min) under supervision of the trained assistants. From the 1941 adolescents who participated, 50 had incomplete data and were not included in the present study. This occurred mainly because these adolescents did not finish the present study’s measures within one class hour. These 50 adolescents were younger than the remaining 1891 adolescents, *t*(1937) = 4.67, *p* < 0.001, *d* = 0.57, and less often enrolled in the highest of the three educational levels, χ^2^(2) = 6.93, *p* = 0.031, Cramer’s V = 0.06, but did not differ significantly in gender composition, χ^2^(1) = 1.54, *p* = 0.214, φ = 0.03.

In the questionnaire adolescents were asked to write a turning point narrative. The majority of adolescents completed this task (*n* = 1580, 83.6%). From those who did not (*n* = 311, 16.4%) some indicated that they could not think of a turning point (59%) or that they did not want to share it (5%), whereas others did not provide a reason (36%). These statements indicated that missing information on narrative characteristics was related to the process of autobiographical narration itself. Writing a turning point narrative or not was not significantly related to age, *t*(1887) = 1.83, *p* = 0.067, *d* = 0.11. Yet, girls were more likely to write a narrative (88.8%) than boys (77.7%), χ^2^(1) = 42.51, *p* < 0.001, φ = 0.15, and the percentages of adolescents who wrote a narrative differed between the lower (76.7%), medium (82.4%), and higher (87.8%) educational levels, χ^2^(2) = 23.72, *p* < 0.001, φ = 0.11. Adolescents who wrote a narrative scored significantly higher on commitment making, *t*(417) = 2.21, *p* = 0.028, *d* = 0.14, identification with commitment, *t*(396) = 2.76, *p* = 0.006, *d* = 0.18, exploration in breadth, *t*(1889) = 5.87, *p* < 0.001, *d* = 0.35, exploration in depth, *t*(420) = 7.01, *p* < 0.001, *d* = 0.45, and ruminative exploration, *t*(1889) = 5.24, *p* < 0.001, *d* = 0.32.^1^ Overall, these findings indicated that turning point narratives were not missing at random. This should be taken into account when interpreting the current findings.

**Table 1:** Illustration of turning point narratives and coding systems

Example turning point narrative	Self-event connection score	Agency score
“I was told that I had to go to a class with a lower educational level, because my grades were too bad. I was very annoyed by this. I realized that school has a big influence on what I can do with my life in the future. I became more serious at school and notice that therefore I have better grades now.”	1	4
“My grandmother passed away. I heard it when I came home from school. I felt sad because my grandfather and I had a good relationship. It changed me in the sense that I know that everyone will die at some point and that I also should expect this a little. Every time when people talk about cancer, I get tears in my eyes. Since then I know that I want to become an oncologist.”	1	2
“My parents were divorced and years later my father got a new girlfriend in another city and we were going to live there. At first I was excited but as we were getting closer to the move it got harder and harder to leave. When I finally moved I went through a hard time and I still find it equally difficult. I see this as a turning point because this is very difficult for me.”	0	0

The examples presented here are composed of parts of narratives by two or three adolescents about a similar event. Additionally, slight changes were made to guarantee anonymity

Analyses focused on the 1580 adolescents (56.2% female) who wrote a turning point narrative. These adolescents were on average 14.7 years old (*SD* = 0.8, range = 12.6–17.4) and enrolled in the lower (18.2%), medium (38.0%), and higher (43.8%) educational levels. Participants were less often enrolled in the lower educational level, compared to the general Dutch population, for which the distribution in the third year of secondary school across these three levels is about 54, 22, and 22% across the lower, medium, and higher level, respectively (Centraal Bureau voor de Statistiek 2016). Information on ethnicity was only requested from a subsample of the Study 1 sample, participating in the longitudinal part of Project-Me (i.e., Study 2). These findings showed that a large majority of adolescents in Project-Me identified themselves as ethnically Dutch.

#### Measures

##### Turning point narratives

Adolescents were asked to write a narrative about an event in which they experienced a turning point in their self-understanding. Turning point narratives were chosen as they have been shown to elicit self-event connections (McLean et al. 2010). The instructions to elicit these turning point narratives were modelled after the adolescent adaptation (McLean et al. 2010) of McAdams’ (2008) instructions. Adolescents were asked to indicate what happened, when it happened, who was involved, what they were thinking and feeling, why the experience was significant, and what it could say about them and their personality. They could use as many words as needed. If adolescents’ responses at least included a topic, their response was judged as a turning point narrative present. Narratives contained on average 121 words (*SD* = 82), ranging from three (e.g., “losing my friend”) to 515 words.

##### Identity commitment and exploration

The Dimensions of Identity Development Scale (DIDS) was used to assess commitment making, identification with commitment, exploration in breadth, exploration in depth, and ruminative exploration with respect to future plans and possible life-paths (Luyckx et al. 2008). Each process was measured with five items to which adolescents could respond using a 5-point scale, ranging from 1 (*strongly disagree*) to 5 (*strongly agree*). Examples of items are “I have plans for what I am going to do in the future” (commitment making), “I sense that the direction I want to take in my life will really suit me” (identification with commitment), “I think about different goals that I might pursue” (exploration in breadth), “I talk with other people about my plans for the future” (exploration in depth), and “I worry about what I want to do with my future” (ruminative exploration). Cronbach’s αs for the five subscales ranged between 0.77 and 0.92.

#### Narrative coding

Narrative coding was performed in three steps, independently for self-event connections and agency. First, using existing coding manuals (cited below), a team of researchers discussed the use of the coding system with a subset of turning point narratives. Based on these discussions, coding manuals were slightly adapted to fit the current data (used coding manuals and full information on adaptations can be retrieved from https://osf.io/tnyaf) and final scores on this initial subset were determined. Second, research assistants were trained using these already coded narratives. Third, the turning point narratives were coded by three (for self-event connections) or two trained coders (for agency), who met regularly (after coding a maximum of 50 narratives) to reach consensus on divergent codes. To prevent divergence in application of the coding manuals (i.e., coder drift; Syed and Nelson 2015), compositions of teams varied throughout the coding process. Examples of coded turning point narratives are shown in Table 1.

##### Self-event connections

Turning point narratives were coded on whether an explicit connection was made between an event and the self (see Pasupathi et al. 2007). Four possible types of connections could be made. First, an event could represent self-stability in two ways: providing an illustrative example of who one is, or is not (i.e., dismiss connection). Second, an event could represent self-change in two ways: an event could have been interpreted to change the self, or to have revealed an aspect of the self. From all coded self-event connections, most involved an event that had changed the self (82%). It was also possible that a turning point narrative did not contain any self-event connection. For the aim of this study, it was only taken into account whether any self-event connection was made (code 1) or none (code 0), which was reliably coded, Fleiss’ κ = 0.71. Most adolescents made one or no self-event connection (91.8%) and only some made two (6.8%) or three (1.5%) self-event connections.

##### Agency

Using the coding manual by Adler et al. (2008), turning point narratives were coded for agency on a 5-point scale. Narratives were coded with a 0 if the participant was described as completely at mercy of circumstances or if they were not written in the first person. A 1 indicated that the participant was somewhat at the mercy of circumstances. A 2 indicated that narratives displayed both agentic and non-agentic elements or lacked information on agency. A 3 indicated that the participant was somewhat agentic. Lastly, a 4 indicated that participants were completely agentic and able to affect their own lives. It was additionally specified that narratives were only rated as non-agentic (0 or 1) if circumstances had a negative influence. This was added because not narrating about one’s role in the course of a positive event was not deemed to indicate a lack of agency (e.g., learning from regular ICT lessons in school that one is interested in this field). Thus, such positive narratives were coded as neutral (2; because of a lack of information) and positive events in which the participant played an active role were coded as agentic (3 or 4; e.g., learning from a self-chosen internship that one is interested in a certain field). Moreover, if a change in agency was described (e.g., first I did not buy a t-shirt because others did not like it, but later I realized it only matters whether I like it and I bought it anyway) the current state of agency was coded. The one-way random intra-class correlation coefficient (ICC) indicated that agency was reliably coded, ICC = 0.78. The final score for agency, which was used in subsequent analyses, consisted of the average score of two independent ratings.

#### Strategy of analysis

In a series of regression analyses it was tested whether self-event connections and agency were significantly associated with each of the commitment and exploration processes. This was tested for self-event connection and agency separately. Additionally, it was tested whether controlling for the length of narratives (i.e., number of words) would alter the findings.

## Results

### Descriptive Statistics

Descriptive statistics of the commitment and exploration processes are available in Table 2. About half of adolescents’ turning point narratives contained at least one self-event connection (45.4%). On average, adolescents’ narratives were slightly agentic (*M* = 2.29, *SD* = 0.90). Adolescents who made a self-event connection scored higher on agency (*M* = 2.56, *SD* = 0.94) than those who did not (*M* = 2.06, *SD* = 0.80), *t*(1410) = 11.14, *p* < 0.001, *d* = 0.57. Turning point narratives that included a self-event connection were significantly longer, *t*(1578) = 9.25, *p* < 0.001, *d* = 0.47. Narrative length was not significantly associated with agency, *r* = 0.02, *p* = 0.537. Furthermore, narrative length was not significantly associated with commitment making, *r* = 0.02, *p* = 0.436, and identification with commitment, *r* = 0.03, *p* = 0.283, but was significantly positively associated with exploration in breadth, *r* = 0.15, *p* < 0.001, exploration in depth, *r* = 0.11, *p* < .001, and ruminative exploration, *r* = 0.10, *p* < 0.001.

**Table 2:** Descriptive statistics and results of the regression analyses in Study 1

Dependent variables:	Commitment making				Identification with commitment				Exploration in breadth				Exploration in depth				Ruminative exploration			
Descriptive statistics *M* (*SD*)	3.39 (0.94)				3.58 (0.72)				3.38 (0.73)				3.20 (0.73)				2.52 (0.79)			
Independent variable	*b*	*p*	*SE*	β	*b*	*p*	*SE*	β	*b*	*p*	*SE*	β	*b*	*p*	*SE*	β	*b*	*p*	*SE*	β
Self-event connections	0.13	0.008	0.05	0.07	0.12	0.001	0.04	0.08	0.19	<0.001	0.04	0.13	0.14	<0.001	0.04	0.09	0.03	0.388	0.04	0.02
Agency	0.06	0.015	0.03	0.06	0.08	<0.001	0.02	0.11	0.04	0.082	0.02	0.04	0.06	0.005	0.02	0.07	−0.04	0.056	0.02	−0.05

Alternative standardized estimates for the binary independent variable self-event connections representing the difference in commitment making, identification with commitment, exploration in breadth, exploration in depth, and ruminative exploration between making a connection or not in standard deviation units of these dual-cycle processes are 0.13, 0.16, 0.26, 0.19, and 0.04, respectively

### Associations Between Dual-Cycle Processes and Narrative Characteristics

Results of the regression analyses are presented in Table 2. Because controlling for narrative length did not substantively affect the strength (Δβ = −0.03) or significance of significant associations, the uncontrolled findings are reported. Adolescents who made a self-event connection scored significantly higher than those without a connection on commitment making (*M* = 3.46, *SD* = 0.92 vs. *M* = 3.33, *SD* = 0.95), identification with commitment (*M* = 3.64, *SD* = 0.71 vs. *M* = 3.53, *SD* = 0.73), exploration in breadth (*M* = 3.48, *SD* = 0.70 vs. *M* = 3.29, *SD* = 0.74), and exploration in depth (*M* = 3.27, *SD* = 0.72 vs. *M* = 3.13, *SD* = 0.74). Making a self-event connection or not was not significantly related to ruminative exploration. Higher agency was significantly associated with higher levels of commitment making, identification with commitment, and exploration in depth. Agency was not significantly associated with exploration in breadth and ruminative exploration.

### Sensitivity Analyses

To examine the robustness of these findings on concurrent associations it was tested whether making alternative decisions in the strategy of analysis would affect the results. First, the regression analyses focused on self-event connections were repeated with a variable that contained the number of made self-event connections instead of the dichotomous variable. This resulted in the same significant associations and no substantial changes in standardized regression coefficients, |Δβ| ≤ 0.006. Second, it was checked whether omitting participants with a neutral score on agency (i.e., 2) because of a lack of information on agency would alter the findings. Performing the regression analyses focused on agency again in this subsample (*n* = 969) resulted in a significant association between agency and ruminative exploration, β = −0.10, *p* = 0.001. Findings on the associations with the other commitment and exploration processes did not change in significance and only slightly in effect size, |Δβ| ≤ 0.036. Lastly, it was checked whether treating the missing turning point narratives as missing at random (i.e., assuming that the missing information on self-event connections and agency can be accounted for by information on age, gender, educational level, and the dual-cycle processes) and applying multiple imputation would alter the findings. Including adolescents without narratives and applying multiple imputation did not alter the significance or effect sizes of the concurrent associations between the narrative characteristics and the dual-cycle processes, |Δβ| < 0.001.

### Summary of Findings

The findings demonstrated that adolescents who made at least one self-event connection were concurrently more strongly committed and more engaged in the adaptive types of exploration (i.e., in breadth and in depth) than those who did not make a self-event connection. Furthermore, a higher degree of narrative agency was associated with being more strongly committed and exploring more in depth. These findings indicated that narrative characteristics are concurrently associated with the processes of the dual-cycle model in adolescence. Nevertheless, associations were small (the highest standardized regression coefficient was 0.13), indicating that the narrative and dual-cycle approach are partly unique in the aspects of identity formation that they capture.

## Study 2

In Study 2, possible predictive effects of narrative characteristics on developments in the processes of the dual-cycle model in adolescence were examined. Characteristics of critical event narratives might foretell how a person changes over time as these might signal strengths that are valuable for the identity formation process, such as having a self-concept rooted in autobiographical stories and viewing the self as agentic. That these strengths are integrated in salient autobiographical narratives might make them especially important for developments in the identity formation process as these narratives are salient and accessible to the individual when entering new situations (Sutin and Robins 2005). Adolescents might rely on memories of previous salient events to guide their actions and when forming new goals (Philippe et al. 2011). If these memories have been constructed in a more adaptive manner (i.e., demonstrating autobiographical reasoning and agency), this might predict more adaptive identity development. Thus, the characteristics of adolescents’ narratives could be used to predict developments in the commitment and exploration processes. Yet, before predicting individual differences, it is important to describe the typical mean-level developmental trends of the commitment and exploration processes

Because identity development becomes a highly salient task in adolescence (Erikson 1950), commitment strength and engagement in exploration are expected to increase from adolescence to adulthood (Luyckx et al. 2013). Overall, the majority of longitudinal studies on commitment and exploration indicated identity maturation between adolescence and adulthood (see Meeus 2016). However, longitudinal studies focusing specifically on adolescence have resulted in mixed findings on mean-level trends of the commitment and exploration processes across several years.

Regarding commitment, previous findings showed mean-level stability (Crocetti et al. 2013; Klimstra et al. 2010) or increases in adolescence (Luyckx et al. 2014). Possibly, average commitment strength was found to remain stable in early and middle adolescence because the process of constructing commitments involves letting go and revising old commitments, such that although change is occurring, it results in mean-level stability. This view fits with previous findings: Studies focused on younger samples all found evidence for mean-level stability (Crocetti et al. 2013; Klimstra et al. 2010). Therefore, it was examined in the current study whether the average stability of commitment strength could be replicated in middle adolescence.

Previous findings on the mean-level developments of the exploration processes in adolescence have been very mixed (Crocetti et al. 2013; Klimstra et al. 2010; Luyckx et al. 2014). Moreover, it is hard to determine what the findings indicate for the three exploration processes separately, because part of the studies focused on a type of exploration (i.e., reconsideration) that likely consists of exploration in breadth and ruminative exploration (Crocetti et al. 2013; Klimstra et al. 2010). Overall, previous findings mostly indicated either mean-level stability or increases in the three exploration processes in middle adolescence. In line with the idea that identity formation becomes a highly salient task in adolescence, it was expected that previous findings which indicated increases in the three types of exploration in middle adolescence could be replicated.

## The Current Study

In Study 2, it was examined whether commitment strength (i.e., commitment making and identification with commitment) remained stable and exploration (i.e., in breadth, in depth, and ruminative) increased on average across middle adolescence. Furthermore, it was examined, as formulated before, whether characteristics of adolescents’ narratives were predictive of developments in the dual-cycle processes. Hypotheses were based on the ideas that making self-event connections is a means of identity formation and that being able to rely on agentic critical event narratives stimulates more adaptive commitment formation and evaluation. Therefore, it was examined whether the presence of self-event connections predicted a more positive increase in commitment strength, and explored whether self-event connections were predictive of changes in the exploration processes. Moreover, it was examined whether a higher degree of agency predicted a more positive increase in commitment strength and adaptive exploration (i.e., increases in exploration in breadth and in depth and decreases in ruminative exploration).

## Method

### Participants and Procedure

Study 2 focused on a subsample of adolescents included in Study 1. One year after the first wave (T1), adolescents could decide to take part in the longitudinal part of Project-Me, which currently consists of two additional waves (T2 and T3). T2 and T3 took place about one and two years after participants’ T1 participation in 2015 and 2016, respectively. Parents were asked to provide consent for their child’s participation in the longitudinal study. They were contacted by asking participants via e-mail to provide parents’ e-mail addresses and by sending out letters via the school if possible. Adolescents and parents who did not respond at T2 were again approached for the data collection of T3. During T2 and T3, adolescents completed the online questionnaire at home and received a small financial compensation of at least €5 for participation and were entered into a lottery to win €50. From the 1,891 adolescents who finished the measures of interest at T1, 270 completed the DIDS at T2 and/or T3.

Yet, 28 of these adolescents had not written a turning point narrative at T1. Adolescents who wrote a narrative at T1 scored significantly higher on exploration in depth at all three waves, *p*s ≤ 0.044, *d*s = 0.43–0.66. Moreover, adolescents who wrote a narrative at T1 scored higher on exploration in breadth at T2 and T3, *p*s ≤ 0.040, *d*s = 0.48–0.59, and higher on commitment making at T3, *p* = 0.034, *d* = 0.55. Descriptive statistics and results on comparisons between adolescents with and without a turning point narrative are presented in Supplemental Table 2 in the Online Resource.

Study 2’s analyses focused on the 242 adolescents who wrote a turning point narrative at T1 and participated at T2 and/or T3. Compared to adolescents who only participated at T1 (i.e., Study 1), adolescents in the longitudinal sample (i.e., Study 2) were more often enrolled in the highest educational level and less often in the lower or medium educational levels, χ^2^(2) = 69.38, *p* < 0.001, Cramer’s V = 0.21, and were more often female, χ^2^(1) = 3.88, *p* = 0.049, φ = 0.05. Moreover, these adolescents scored higher on agency within their turning point narratives than unselected participants, *t*(1578) = 3.79, *p* < 0.001, *d* = .27, and more often made a self-event connection, χ^2^(1) = 16.76, *p* < 0.001, φ = 0.10. Adolescents only included in Study 1 did not differ significantly from those included in Study 2 in age and the commitment and exploration processes, *p* ≥ 0.086.

Adolescents (62.0% female) in the Study 2 sample were on average 14.7 years at T1 (*SD* = 0.7, range = 13.0–16.7). At T1, they were enrolled in the lower (7.0%), medium (25.2%), or higher (67.8%) educational level. The majority (95.0%) identified themselves as ethnically Dutch. Adolescents had completed the DIDS at T2 (72.3%) and/or at T3 (68.2%). Little’s (1988) Missing Completely at Random (MCAR) test on the study variables was not significant, χ^2^(22) = 11.39, *p* = 0.969, indicating that missing values in the DIDS at T2 or T3 occurred completely at random. Participants with missing data were included in Mplus 7 using Full Information Maximum Likelihood (FIML; Muthén and Muthén 1998–2015).

### Measures

Like Study 1, Study 2 focused on the measurements of the DIDS and adolescents’ turning point narratives at T1. In addition, Study 2 focused on measurements of the DIDS at T2 (αs = 0.77–0.94) and T3 (αs = 0.79–0.95). Prior to examining the longitudinal development in the mean-levels of commitment and exploration, longitudinal measurement invariance of the dual-cycle processes was examined (Widaman et al. 2010). Longitudinal Confirmatory Factor Analysis models estimated in Mplus 7 (Muthén and Muthén 1998–2015) showed evidence for scalar invariance across the three waves for all five processes. Scalar invariance refers to similarity of the construct (invariant factor loadings) as well as similarity of the levels of the underlying items (invariant intercepts) across time (Van de Schoot et al. 2012). Moreover, for the models of commitment making, identification with commitment, and ruminative exploration, strict invariance was found, and for the models of exploration in breadth and in depth partial strict invariance was found. These findings thus indicated that it was appropriate to create the composite mean scores of the Dutch DIDS subscales to examine developmental trends across middle adolescence (Steinmetz 2013), and fit with previous findings on the Greek version of the DIDS (Mastrotheodoros and Motti-Stefanidi 2017). Full information on the tests of longitudinal measurement invariance is available in the Online Resource (see Supplemental Table 3).

### Strategy of Analysis

First, changes in mean-levels of the dual-cycle processes across the three waves were examined with Latent Growth Curve (LGC) modeling in Mplus 7 (Muthén and Muthén 1998–2015) with a Robust Maximum Likelihood estimator (MLR; Satorra and Bentler 2001). Based on individuals’ growth trajectories, LGC modeling estimates a mean level (intercept) and the mean change rate (slope). To take individual differences in the time between waves into account, latent growth curves were estimated based on the exact time of each of the waves using the TSCORES option (Coulombe et al. 2016). Thus, intercepts reflect the estimated level of a process at T1 and slopes reflect changes across one year. The latent growth curves for all five processes were estimated in one model in order to appropriately handle the missing data using FIML. The model included associations between the five intercepts and between the five slopes. Next, it was tested whether self-event connections and agency predicted changes in the dual-cycle processes over time by alternately including them as predictors of the intercepts and slopes. In predicting the slopes, participants’ starting points on the specific processes were added as control variables (i.e., each slope was regressed on the corresponding intercept). Lastly, it was checked whether adding narrative length as predictor of the intercepts and slopes altered the findings.

## Results

### Descriptive Statistics

Of the adolescents in Study 2, 57.4% made at least one self-event connection in their turning point narrative at T1. Adolescents’ mean agency score was 2.49 (*SD* = 0.89). Making a self-event connection was associated with higher narrative agency (*M* = 2.75, *SD* = 0.82) than not making a connection (*M* = 2.14, *SD* = 0.85), *t*(240) = 5.68, *p* < 0.001, *d* = 0.74. Narratives with a self-event connection were significantly longer, *t*(240) = 3.01, *p* = 0.003, *d* = 0.39. Agency was also positively associated with narrative length, *r* = 0.15, *p* = 0.018. Descriptive statistics of the dual-cycle processes across the three waves are presented in Table 3. Associations between self-event connection and agency at T1 and the dual-cycle processes across the three waves are shown in Supplemental Tables 4 and 5.

**Table 3:** Descriptive statistics and results of the latent growth curve model of the dual-cycle processes in Study 2

	Descriptive Statistics			Growth factors			
	T1	T2	T3	Intercept		Slope	
	*M* (*SD*)	*M* (*SD*)	*M* (*SD*)	*M*	σ^2^	*M*	σ^2^
Commitment making	3.48 (0.89)	3.45 (1.00)	3.50 (0.97)	3.47***	0.57***	0.01	0.15***
Identification with commitment	3.65 (0.73)	3.62 (0.73)	3.56 (0.76)	3.65***	0.35***	−0.04	0.11***
Exploration in breadth	3.42 (0.76)	3.64 (0.72)	3.61 (0.68)	3.44***	0.30***	0.10***	0.04**
Exploration in depth	3.27 (0.69)	3.41 (0.73)	3.46 (0.70)	3.27***	0.23***	0.10***	0.08***
Ruminative exploration	2.53 (0.77)	2.71 (0.80)	2.81 (0.93)	2.53***	0.34***	0.15***	0.08***

A Latent Growth Curve Model on a sample that included also the 28 adolescents that participated in the longitudinal part of the study but did not write a turning point narrative at T1 resulted in the same findings

**p < 0.01; ***p < 0.001

### Mean-Level Changes in Dual-Cycle Processes

Results of the LGC model (see Table 3) showed that, as predicted, the mean-levels of commitment making and identification with commitment did not significantly change over time. In contrast, and as predicted, the mean-levels of exploration in breadth, exploration in depth, and ruminative exploration all increased significantly across the two years. Moreover, findings showed significant variance between adolescents in the slopes of all five dual-cycle processes, indicating that the development of these processes over time differed significantly between adolescents.

**Fig. 1:**
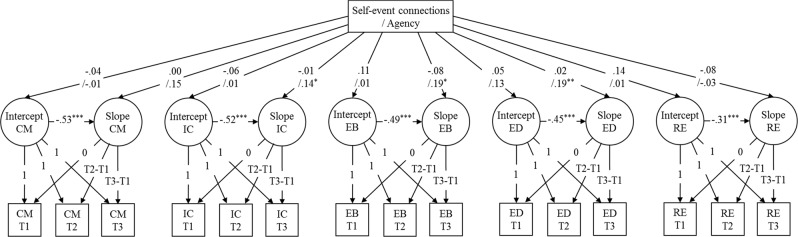
Results from two latent growth curve models with either self-event connections or agency included as predictor of the intercepts and slopes of the commitment and exploration processes in Study 2. CM = Commitment making; IC = Identification with commitment; EB = Exploration in breadth, ED = Exploration in depth; RE = Ruminative exploration. Correlations between the five intercepts and correlations between the five slopes are not shown, but were included in the tested models. Coefficients before the slash indicate predictions by self-event connections. Coefficients after the slash represent predictions by agency. Numbers are standardized regression coefficients representing the difference in y in y standard deviation units for a standard deviation change in x (i.e., StdYX). For the binary self-event connection variable the standardized estimates representing the difference in y in y standard deviation units between no or at least one self-event connection (i.e., StdY) are −0.07, −0.13, 0.23, 0.10, and 0.29 for the intercepts and 0.00, −0.02, −0.17, 0.05, and −0.16 for the slopes of CM, IC, EB, ED, and RE, respectively. Unstandardized estimates, standard errors, and *ps* are displayed in Supplemental Tables 6 and 7 in the Online Resource. *p < 0.05; **p < 0.01; ***p < 0.001

Prior to testing predictive effects of the narrative characteristics, each slope was regressed on the corresponding intercept. The relatively lower Akaike’s Information Criterion (AIC) and Bayesian Information Criterion (BIC) indicated that this model (AIC = 5656, BIC = 5865) fitted the data better than alternative models (Byrne 2013) without these regression coefficients (AIC = 5709, BIC = 5901) or with correlations instead of regression coefficients (AIC = 5717, BIC = 5926). Intercepts were significantly negatively related to the slopes (see Fig. 1), indicating that adolescents who scored higher on a specific dual-cycle process increased less in this specific process over time compared to those who scored lower on this process. By including these moderate to strong negative associations, other predictions on the slopes were controlled for individual differences in the possibility to increase in commitment strength and exploration.

### Predicting Individual Changes in Commitment and Exploration

Next, self-event connections and agency were alternately added to the LGC model as predictors of the intercepts and slopes of the commitment and exploration processes. Because controlling for narrative length did not alter the findings, the uncontrolled findings are reported (see Fig. 1 and Supplemental Tables 6 and 7). Narrative length only had a borderline significant association with the intercept of exploration in breadth, β = 0.14, *p* = 0.050, and was not associated with any of the other intercepts or with any of the slopes, *p* ≥ 0.104.

Whether or not adolescents’ turning point narratives contained a self-event connection at T1 was not a significant predictor of any of the intercepts or slopes of the commitment and exploration processes. Agency was not significantly associated with any of the intercepts of the commitment and exploration processes, or with the slopes of commitment making and ruminative exploration. However, a higher degree of agency at T1 did predict a significantly more positive slope of identification with commitment, β = 0.14, *p* = 0.022, exploration in breadth, β = 0.19, *p* = 0.027, and exploration in depth, β = 0.19, *p* = 0.009. This shows that adolescents who expressed more agency in their turning point narrative increased more in identification with commitment, exploration in breadth, and exploration in depth over time, relative to adolescents who expressed less agency.

### Sensitivity Analyses

To examine the robustness of the findings on the predictive effects of narrative characteristics on dual-cycle processes it was tested whether making alternative decisions in the strategy of analysis would affect the results. First, repeating the LGC analysis with a variable containing the number of made self-event connections instead of a dichotomous variable showed again that self-event connections were not significantly associated with the intercepts and slopes of the dual-cycle processes, *p* ≥ 0.283. Second, it was tested whether excluding participants with a neutral score on agency (i.e., 2) because of a lack of information on agency would alter the findings. Repeating the LGC analyses on the predictive effects of agency in this subsample (*n* = 164) showed that this time agency failed to significantly predict the slopes of identification with commitment, β = 0.13, *p* = 0.070, and exploration in depth, β = 0.14, *p* = 0.065. Yet, agency was still significantly associated with the slope of exploration in breadth, β = 0.26, *p* = 0.012. Moreover, agency was still not a significant predictor of the slopes of commitment making and ruminative exploration or the intercepts of the five dual-cycle processes, *p* ≥ 0.120.

Lastly, it was checked whether treating the missing turning point narratives as missing at random and applying multiple imputation (i.e., missing information on self-event connection and agency was imputed based on age, gender, educational level, and the dual-cycle processes) would alter the findings. In order to apply multiple imputation, LGC models were estimated based on fixed one-year intervals instead of individually-varying times of T2 and T3. This specific change resulted in a predictive effect of agency on the slope of commitment making that was on the borderline of significance, β = 0.16, *p* = 0.050, but no other changes. The findings based on multiple imputation showed that agency significantly positively predicted the slope of commitment making, β = 0.16, *p* = 0.038, but failed to significantly predict the slope of exploration in breadth, β = 0.19, *p* = 0.155, in contrast to the present study’s main findings. Other findings remained the same. Agency significantly predicted the slopes of identification with commitment, β = 0.18, *p* = 0.017, and exploration in depth, β = 0.24, *p* = 0.020, but not ruminative exploration or any of the intercepts, *p* ≥ 0.138. Self-event connections was not significantly associated with any of intercepts or slopes of the dual-cycle processes, *p* ≥ 0.158.

Overall, these findings demonstrate that narrative agency was also in alternative analyses significantly predictive of developments in the dual-cycle processes, yet the specific dual-cycle process in which agency predicted developments was not consistent. Because these alternative analyses are considered suboptimal (i.e., decreases in power and treating not randomly missing variables as randomly missing) their findings regarding specific associations are likely less reliable than the main analyses. Yet, they do show the importance of future replications of the predictive effects of agency on developments in the dual-cycle processes.

### Summary of Findings

The findings indicated that on average commitment strength remained stable in middle adolescence, while all three exploration processes increased. The concurrent associations of self-event connections and agency with the dual-cycle processes as shown in the large cross-sectional sample of Study 1 could not be replicated in the smaller subsample of Study 2. This was likely caused by a lack of power to detect small associations. That is, although a sample size of 242 provides sufficient power (i.e., 0.80) to find associations (i.e., correlations) of 0.18 and stronger, this sample size is insufficient to find smaller effects (e.g., power = 0.34 when *r* = 0.10, α = 0.05, two-tailed; Faul et al. 2007). Although the presence of self-event connections was not predictive of individual differences in developments of the dual-cycle processes, agency was. Adolescents who expressed more agency in their narrative not only showed slightly more identification with commitment and exploration in depth concurrently (i.e., Study 1), but also increased more in these dual-cycle processes over time. This positive prospective effect was also apparent for exploration in breadth. These findings show that narratives’ motivational theme can foretell developments in the dual-cycle processes.

## Discussion

The narrative and the dual-cycle approach are two commonly used approaches to study identity formation. Although both approaches originated from Erikson’s writings and are thought to be complementary (McLean and Pasupathi 2012), their empirical links have rarely been examined in adolescence and across time. This is unfortunate because knowledge on their links could provide information on the degree to which the approaches overlap and on how both approaches can inform each other. In the present research, concurrent and prospective links between key dimensions of both approaches were examined in adolescence.

Overall, the current findings show that the narrative characteristics of making a self-event connection and agency are concurrently positively associated with commitment strength and more adaptive exploration. Yet, these associations were small. In addition, the degree of agency in adolescents’ narratives foretold future developments of the dual-cycle processes. Specifically, narrating about oneself as agentic predicted increases in identification with commitment and the more adaptive exploration processes of exploring in breadth and in depth.

### Mean-Level Developmental Trends of Dual-Cycle Processes

When examining how narrative characteristics are linked to and predictive of adolescents’ commitment and exploration, it is important to realize what the average developmental trends of these processes are. The finding that commitment strength remained on average stable in middle adolescence replicated most previous findings (Crocetti et al. 2013; Klimstra et al. 2010). Likely, this average stability is caused by interindividual heterogeneity, which was reflected in significant variance between adolescents’ slopes and has previously been shown in studies on identity formation trajectories (Meeus et al. 2012). Being engaged in identity formation not only involves establishing strong commitments, but also questioning current commitments.

Furthermore, the present findings showed that in middle adolescence, engagement in exploration in breadth, exploration in depth, and ruminative exploration on average increased. Previous findings have been rather mixed, but some also suggested increases (Crocetti et al. 2013; Klimstra et al. 2010; Luyckx et al. 2014). The present findings are consistent with the existing ideas within the dual-cycle approach (e.g., Luyckx et al. 2013) and narrative approach (e.g., Habermas and Reese 2015) that identity is a salient task in adolescence, in which adolescents generally increasingly engage.

Although not exactly in line with how the processes are presented in the dual-cycle model by Luyckx et al. (2006), the findings on average stability of commitment strength and increases in exploration do fit with the general idea of dual-cycle models that identity development generally starts with a phase of exploring possibilities (i.e., identity formation; Crocetti et al. 2008; Luyckx et al. 2006). Adolescents examine which possibilities exist, including the ones they currently have in mind. This likely precedes a phase in which made commitments are strengthened (i.e., identity evaluation). Nevertheless, adolescents differed in how the five processes changed over time, which further emphasized that it is worthwhile to examine different trajectories (Meeus et al. 2012) and how individual variations in developments of the commitment and exploration processes can be predicted.

### Integrating Narrative and Dual-Cycle Model Approaches

#### Self-event connections and the dual-cycle model

Overall, the findings indicated that autobiographical reasoning was positively related to adaptive processes of the dual-cycle model. Specifically, adolescents who made a self-event connection were more strongly committed and explored more in breadth and in depth, but did not engage more in ruminative exploration. Yet, the significant associations with the dual-cycle processes were small and (likely because of this) non-significant when re-examined in the smaller subsample of Study 2. Moreover, making self-event connections was not predictive of increases in commitment strength. The present findings thus showed that the processes of narrating a coherent life story and pursuing strong commitments are linked, but that information from each of the approaches provides complementary knowledge on adolescents’ identity formation.

Although making self-event connections and the formation and evaluation of identity commitments are both focused on how individuals establish a sense of personal continuity, both approaches primarily focus on a different element of this personal continuity. That is, whereas self-event connections mostly provide personal continuity between the past and the present (e.g., flunking a grade has made one more conscientious at school), commitments mostly provide personal continuity between the present and the future (e.g., the commitment to becoming a teacher provides current certainty about one’s future self; McLean and Pasupathi 2012). Therefore, individuals who have constructed self-event connections as well as strong commitments might experience most personal continuity.

Furthermore, in addition to indicating engagement in the construction of a coherent narrative identity, the presence of a self-event connection reflects an ability to construct these connections (McLean et al. 2019). In contrast, a high degree of identity exploration in breadth and in depth indicates engagement in relatively adaptive exploration types, but not whether this has led to meaningful outcomes. This difference in emphasis on the outcome between the two processes could explain the small associations between them in the present study on adolescents and previous studies among young adults (McLean and Pratt 2006; McLean et al. 2016; Merrill et al. 2016). In light of these findings, future studies could test whether the dual-cycle exploration processes are more strongly linked to narrative characteristics that focus more specifically on engagement in autobiographical reasoning, such as the presence of exploratory processing as used in adult samples (Pals 2006).

Moreover, the differences in the methodologies of the narrative and dual-cycle approach could explain the small associations. In the narrative approach, adolescents report narratives, which are coded for autobiographical reasoning by others (i.e., trained coders). In contrast, in the dual-cycle approach adolescents report their own view on the extent to which they engage in a process. These differences in methods could, by themselves, account for the small association. For example, it is possible that adolescents who often engage in connecting events to their self-concept, without having to put a lot of effort in this, do not view themselves as highly engaged in identity exploration. Alternatively, it is possible that there is less effort put into reporting processes on a scale, than in autobiographical narrating and people report that they are more engaged in such processes than they actually are. The small associations between self-event connections and the dual-cycle processes could thus reflect differences in the methodologies of the two approaches.

#### Agency and the dual-cycle model

It was hypothesized that viewing oneself as agentic and being able to rely on critical event narratives in which the self was agentic would stimulate adaptive formation and evaluation of commitments. The present findings confirmed this, as a higher degree of agency in adolescents’ narratives was significantly, but weakly, related to being more strongly committed and engaging more in exploration in depth. Consistent with their small effect size, these associations disappeared when re-examined in the smaller subsample of Study 2. A possible explanation for the small associations between narrative agency and commitment strength and exploration in depth is that adolescents develop their identity within a socio-cultural context. While some adolescents might receive a lot of support and opportunities for identity development, others might be confronted with contextual barriers (Yoder 2000). Consequently, even adolescents who describe themselves as highly agentic within their autobiographical stories might not be able to commit to the identity options of their choice. Such contextual factors might reduce the strength of associations between narrative agency and the dual-cycle processes. In addition, it might take time for narrative agency to affect adolescents’ dual-cycle processes.

The longitudinal findings showed that adolescents who expressed more agency in their narrative did increase more in identification with commitment, exploration in breadth, and exploration in depth over time, compared to adolescents who lacked this narrative agency. This suggests that believing that one is agentic and being able to rely on critical event narratives in which one is agentic stimulates adolescents to commit to options that they see fit them best and might encourage the more normative trajectory of exploration in adolescence. These findings, if replicated, might indicate a promising way to promote the formation of strong commitments and adaptive identity exploration among adolescents, as previous findings showed that agency as displayed in personal narratives can be increased through interventions (Adler 2012).

#### No narrative and the dual-cycle model

While executing the present research, it was evident that a particular group of adolescents did not narrate a turning point. Most interestingly, these adolescents had slightly weaker commitments and engaged slightly less in all three exploration processes compared to adolescents who did narrate a turning point. Moreover, the small sample of adolescents who did not narrate a turning point in the longitudinal subsample later engaged consistently relatively less in exploration in breadth and in depth, and reported two years later significantly lower commitment making. Although adolescents who did not narrate a turning point might have different reasons for this, reluctance or inability to share an autobiographical story does seem to indicate less engagement in the formation and evaluation of identity commitments. This fits with the idea that engaging in autobiographical narration is a way of exploring identity commitments (McLean and Pasupathi 2012), and underscores that the two identity formation approaches are linked.

That said, the lack of information on autobiographical narrating by these adolescents likely suppressed the associations between narrative characteristics and the dual-cycle processes. For instance, some of the adolescents who did not share a turning point might have been unable to come up with such a narrative and were thus unable to make a self-event connection. Thus, part of the associations between autobiographical reasoning and the dual-cycle processes might have been evident in the lower commitment and exploration scores of adolescents without a narrative, resulting in weaker associations between self-event connections and commitment strength and exploration.

One could argue that this type of missing data is specific for written narratives, because here no researcher is present to encourage narration. Yet, it is likely that adolescents who did not *want* to write down a turning point narrative also would not want to participate in a life story interview. Nevertheless, when collecting written narratives, it remains unclear how different reasons for not sharing a turning point are related to the dual-cycle processes. In the current study, a large subgroup of adolescents did not provide information on why they did not narrate. Therefore, it is recommended that future studies add follow-up questions after the narrative prompt on why adolescents might not write a narrative and examine their engagement in the commitment and exploration processes concurrently and over time.

### Strengths and Limitations

Strengths of the present research were the combined use of a large cross-sectional sample (Study 1) and a longitudinal subsample (Study 2). The large cross-sectional sample made it possible to detect even small associations between the two identity formation approaches. The longitudinal subsample made it possible to examine whether both approaches were also linked when predicting adolescents’ developments in commitment and exploration. Another strength was the use of two different methods to study identity formation: a self-report questionnaire and coded narrative data. This reduces the chance that associations were resulting from shared measurement error.

There were, however, also limitations that need to be acknowledged. First, the longitudinal subsample in Study 2 was relatively small and not completely representative of the larger sample from which it was derived. Adolescents in the subsample made more often a self-event connection and expressed more agency. Yet, these differences were small and the subsample differed not significantly in the dual-cycle processes, indicating that biases might be relatively minor. Nevertheless, because of these limitations it is especially important for future research to test whether the present longitudinal findings can be replicated.

Second, the present research focused on the characteristics of one autobiographical narrative. Previous findings showed high intra-individual variability in the characteristics of different types of narratives (McLean et al. 2016). Choosing an appropriate narrative prompt is thus important. Turning point prompts are known to elicit self-event connections (McLean et al. 2010) and were thus deemed appropriate for the present research. Yet, assessing the characteristics in future studies across multiple narratives or a full life story interview (McAdams 2008) might be more reliable and could result in stronger associations with the dual-cycle processes.

Third, the present research focused on prospective effects between the narrative and the dual-cycle approach, but only in one direction. Another possible way of linking the two approaches would be to examine whether the dual-cycle processes are predictive of developments in autobiographical narration. Apart from the fit of the examined direction with the literature (McLean and Pasupathi 2012), prospective effects in this other direction were not examined in the present research because of insufficient data. That is, the not at random missing data on the turning point narratives would make it difficult to accurately estimate changes in the narrative characteristics. Future research could take this into account during data collection, for example by collecting more measurement waves, and examine whether adolescents’ engagement in the dual-cycle processes predicts changes in the characteristics of their autobiographical narratives over time.

Fourth, it should be pointed out that the present research focused on middle adolescence. Although this was rather novel and adolescence is a key period to examine identity formation (Erikson 1968), the present findings might not generalize to other periods in the human life span. Middle adolescence could be too early to foretell individual differences in the development of commitment strength based on the degree of self-event connections, as a coherent narrative identity takes time to develop (Habermas and Reese 2015). Future research should examine whether autobiographical reasoning is predictive of developments in commitment and exploration in other age periods, such as young adulthood.

Fifth, the present research was limited to adolescents in the Netherlands. This did extend previous studies on links between autobiographical narration and commitment and exploration in other Western cultures (Carlsson et al. 2015; Glavan et al. 2019; McLean and Pratt 2006; McLean et al. 2016). Yet, young people from non-Western cultures might differ in how they costruct their narrative identity (e.g., Reese et al. 2014), which might result in different associations between the two identity formation approaches. Future studies might examine how the two approaches relate to each other in non-Western cultures.

## Conclusion

The narrative and the dual-cycle approach capture identity formation in two different ways. Although these approaches have been thought to be complementary, their empirical links have only scarcely been investigated. Knowledge on their empirical links in adolescence and across time has been especially lacking. Therefore, the present research examined concurrent and prospective links between key dimensions of both approaches in adolescence. The findings provided support for concurrent links. Adolescents who showed autobiographical reasoning and agency in their autobiographical narrating were more engaged in the commitment and adaptive exploration processes of the dual-cycle model. However, these associations were relatively weak, consistent with previous studies in young adulthood. This indicates that both approaches primarily capture unique aspects of the identity formation process and could thus complement each other. An example of this was the finding that a higher degree of agency in one key autobiographical narrative foretold a steeper increase in identification with commitments and adaptive exploration in middle adolescence. This shows that by paying attention to adolescents’ autobiographical stories one can not only get to know their current narrative identity, but also more accurately foretell their engagement in exploring and strengthening commitments.

## Supplementary Information

Supplementary Information.

## Footnotes

1 Supplemental Table 1 in the Online Resource provides the descriptive statistics of the commitment and exploration processes across groups of adolescents with different reasons or no provided reason for not writing a turning point narrative.
