# Supplementary material for: Adolescents’ Identity Formation: Linking the Narrative and the Dual-Cycle Approach
Source: J Youth Adolesc. 2019 Aug 12;49(4):818–35. doi: 10.1007/s10964-019-01096-x (PMC7105420; doi:10.1007/s10964-019-01096-x)
Supplement: Supplementary file 1 — Supplementary Information. [file 10964_2019_1096_MOESM1_ESM.pdf]

Online Resource by

Adolescents' Identity Formation:

Linking the Narrative and the Dual-Cycle Approach

Lotte van Doeselaar, Kate C. McLean, Wim Meeus, Jaap J. A. Denissen, & Theo A. Klimstra

Journal of Youth and Adolescence

Table S1

Descriptive Statistics of the Commitment and Exploration Processes Across Groups of Adolescents Differing in Their Reason for not Writing a Turning Point Narrative (Study 1)

	Commitment making	Identification with commitment	Exploration in breadth	Exploration in depth	Ruminative exploration
	M (*SD*)	*M* (*SD*)	*M* (*SD*)	*M* (*SD*)	*M* (*SD*)
Wrote a narrative	3.39 (0.94)	3.58 (0.72)	3.38 (0.73)	3.20 (0.73)	2.52 (0.79)
No narrative	3.25 (1.03)	3.43 (0.88)	3.11 (0.82)	2.85 (0.80)	2.26 (0.79)
Don't want to share	3.45 (1.12)	3.39 (0.97)	3.30 (0.96)	3.08 (0.83)	2.29 (0.75)
Don't know	3.24 (0.99)	3.47 (0.81)	3.13 (0.73)	2.87 (0.75)	2.29 (0.80)
No clear reason	3.25 (1.10)	3.38 (0.97)	3.04 (0.92)	2.79 (0.87)	2.22 (0.80)

Note. From the adolescents who did not write a turning point narrative (n = 311, 16.4% of total sample), 16 stated that they did not want to share one, 184 stated that they could not think of a turning point event, and 111 did not provide a clear reason. Adolescents were not asked to provide a reason when they did not write a turning point narrative.

Table S2

Descriptive Statistics for Adolescents in the Longitudinal Sample without a Turning Point Narrative at T1 and Comparisons with the Study 2 Sample (with a Turning Point Narrative at T1)

	T1		T2		T3	
	M (*SD*)	*d*	*M* (*SD*)	*d*	*M* (*SD*)	*d*
Commitment making	3.23 (1.13)	.25	3.32 (1.24)	.12	2.99 (0.89)	$.55^{*}$
Identification with commitment	3.46 (0.90)	.23	3.31 (0.83)	.40	3.27 (0.65)	.41
Exploration in breadth	3.15 (0.95)	.32	3.27 (0.80)	$.48^{*}$	3.23 (0.58)	$.59^{*}$
Exploration in depth	2.81 (0.68)	$.66^{**}$	3.04 (0.96)	$.43^{*}$	3.00 (0.71)	$.65^{**}$
Ruminative exploration	2.42 (0.77)	.14	2.84 (0.72)	.17	2.97 (0.82)	.17

Note. Differences between adolescents with (Study 2 sample) and without (descriptive statistics shown here) a turning point narrative at T1 in the five identity processes across time were tested with t-tests. For the comparisons at T1, T2, and T3 the n of adolescents without a turning point narrative at T1 was 28, 19, and 18, respectively. If significant, adolescents without a turning point narrative at T1 scored lower (see Table 3).
$^{*} p < .05$; $^{**} p < .01$.

Measurement Invariance

Longitudinal measurement invariance of the DIDS subscales was tested following steps described by Widaman, Ferrer, and Conger (2010). A series of longitudinal Confirmatory Factor Analysis (CFA) models was estimated in Mplus 7 (Muthén & Muthén, 1998-2015). Each dual-cycle process was tested separately. For reasons of slightly non-normally distributed variables, a Full Information Robust Maximum Likelihood estimator (MLR) was used (Satorra & Bentler, 2001). Model fit was considered to be acceptable when the Comparative Fit Index (CFI) was above 0.90 and the Root Mean Square Error of Approximation (RMSEA) was below 0.08 (Byrne, 2013). A significant reduction in model fit was concluded if two of the following three criteria were met: $\Delta\chi^2_{SB}$ significant at < .050, ΔCFI < -.010, and ΔRMSEA > .015 (Chen, 2007; Cheung & Rensvold, 2002).

First, it was examined whether a longitudinal Confirmatory Factor Analysis (CFA) model fitted the data sufficiently (Widaman et al., 2010). A sufficient model fit would indicate configural invariance, which refers to the validity of the same CFA at every wave (Van de Schoot, Lugtig, & Hox, 2012). In addition to CFA models for one of the identity processes at all three waves, longitudinal CFA models contained covariances between the latent variables and between the residuals of like items across the waves. Only the model for exploration in breadth did not fit the data well, χ^2_{SB} (72) = 421.88, $p < .001$, CFI = .68, RMSEA = .14. The Modification Indices indicated that the residuals of the items “I am considering a number of different lifestyles that might suit me” and “I am thinking about different lifestyles that might be good for me” were correlated within every wave. Correlating the residuals of these items was deemed reasonable, as these items were more alike than others. Including these correlations resulted in an acceptable model fit for the model of exploration in breadth. The fit statistics of all models are displayed in Table S2.

Second, metric invariance was tested by constraining all factor loadings of like items to be equal across time. For none of the identity processes this resulted in a significantly worse model fit (see Table S2). Third, scalar invariance was tested by also constraining all intercepts of like items to be equal across time. Again, this did not result in a significantly worse model fit for any of the commitment and exploration processes (see Table S2). Moreover, all final models in which factor loadings and intercepts of like items were constrained to be equal across time had a sufficient model fit.

Lastly, it was checked whether constraining strict invariance would decrease the model fit by constraining the residuals of corresponding items to be time invariant. Adding these constraints did not significantly worsen the model fit for the model of commitment

making, identification with commitment, and ruminative exploration (see Table S2). However, the model fit for the model of exploration in breadth, $\Delta\chi^2_{SB}$ (10) = 22.09, p = .015, ΔCFI = -.011, ΔRMSEA = .000, and exploration in depth, $\Delta\chi^2_{SB}$ (10) = 25.09, p = .005, ΔCFI = -.021, ΔRMSEA = .007, became significantly worse. Next, it was checked for both models for which item constraining the residuals resulted in the biggest decrease in model fit, and subsequently which wave resulted in the biggest decrease in model fit. Based on these findings, a model was tested in which the residuals of four items were constrained across time and the residual of one item was constrained across two waves. The residual of this latter item was freely estimated at one wave. For exploration in breadth this was the item “I think about different goals that I might pursue” at T3. For exploration in depth this was the item “I think about whether the aims I already have for life really suit me” at T2. These models with partial strict invariance did not fit significantly worse than the models with scalar invariance (see Table S2).

Table S3

Model Fit of Longitudinal CFA Models, Used to Test Longitudinal Measurement Invariance (Study 2)

		Model fit					Change in model fit [a]				
Identity process	Model	χ^2_{SB}	*df*	*p*	CFI	RMSEA	$\Delta\chi^2_{SB}$	*df*	*p*	ΔCFI	ΔRMSEA
Commitment making	1. Configural invariance	103.40	72	.009	.982	.042					
	2. Metric invariance	111.57	80	.011	.982	.040	6.44	8	.598	.000	-.002
	3. Scalar invariance	120.83	88	.012	.981	.039	8.87	8	.353	-.001	-.001
	4. Strict invariance	149.07	98	.001	.971	.046	22.65	10	.012	-.010	.007
Identification with commitment	1. Configural invariance	179.78	72	< .001	.924	.079					
	2. Metric invariance	192.24	80	< .001	.921	.076	12.64	8	.125	-.003	-.003
	3. Scalar invariance	202.49	88	< .001	.919	.073	9.29	8	.318	-.002	-.003
	4. Strict invariance	218.88	98	< .001	.915	.071	16.69	10	.082	-.004	-.002
Exploration in breadth	1. Configural invariance [b]	155.27	69	< .001	.921	.072					
	2. Metric invariance	163.16	77	< .001	.921	.068	7.28	8	.507	.000	-.004
	3. Scalar invariance	176.43	85	< .001	.916	.067	12.87	8	.116	-.005	-.001
	4. Partial strict invariance	192.37	94	< .001	.910	.066	16.30	9	.061	-.006	-.001
Exploration in depth	1. Configural invariance	98.18	72	.022	.967	.039					
	2. Metric invariance	102.48	80	.046	.972	.034	4.75	8	.784	.005	-.005
	3. Scalar invariance	117.45	88	.020	.963	.037	15.61	8	.048	-.009	.003
	4. Partial strict invariance	132.74	97	.009	.955	.039	14.96	9	.092	-.008	.002
Ruminative exploration	1. Configural invariance	124.08	72	< .001	.937	.055					
	2. Metric invariance	132.66	80	< .001	.937	.052	7.48	8	.486	.000	-.003
	3. Scalar invariance	140.31	88	< .001	.937	.050	6.65	8	.575	.000	-.002
	4. Strict invariance	153.47	98	< .001	.933	.048	13.23	10	.211	-.004	-.002

Note. [a] The change in model fit refers to a comparison with the model in the previous line.
[b] The model of exploration in breadth included correlations between the residuals of two pairs of items within every wave, as described in the text.

Table S4

Comparisons between Adolescents With and Without a Self-event Connection at T1 in the Commitment and Exploration Processes Across the Three Waves (Study 2)

	DIDS T1			DIDS T2			DIDS T3		
Self-event connection T1:	Yes	No		Yes	No		Yes	No	
	M (*SD*)	*M* (*SD*)	*d*	*M* (*SD*)	*M* (*SD*)	*d*	*M* (*SD*)	*M* (*SD*)	*d*
Commitment making	3.46 (0.90)	3.51 (0.88)	.06	3.38 (1.03)	3.54 (0.94)	.16	3.55 (1.00)	3.43 (0.94)	.11
Identification with commitment	3.61 (0.74)	3.70 (0.71)	.13	3.60 (0.71)	3.65 (0.76)	.06	3.56 (0.79)	3.55 (0.71)	.01
Exploration in breadth	3.48 (0.72)	3.34 (0.81)	.19	3.67 (0.71)	3.59 (0.74)	.11	3.60 (0.70)	3.62 (0.66)	.03
Exploration in depth	3.29 (0.72)	3.24 (0.65)	.07	3.45 (0.72)	3.36 (0.75)	.12	3.49 (0.75)	3.42 (0.61)	.10
Ruminative exploration	2.60 (0.76)	2.43 (0.77)	.23	2.77 (0.78)	2.64 (0.82)	.15	2.77 (0.94)	2.88 (0.92)	.11

Note. DIDS = Dimensions of Identity Development Scale. Differences between the two groups were tested with *t*-tests, but were not significant, $p \geq .079$.

Table S5

Correlations between Agency at T1 and the Commitment and Exploration Processes across the Three Waves (Study 2)

	Agency T1		
	DIDS T1	DIDS T2	DIDS T3
Commitment making	.00	.04	.13
Identification with commitment	.02	.06	.15*
Exploration in breadth	.01	.13	.13
Exploration in depth	.10	.19*	.19*
Ruminative exploration	.01	.02	.00

Note. DIDS = Dimensions of Identity Development Scale.

* $p < .05$

Table S6

Results of the Latent Growth Curve Model with Self-event Connection as Predictor of the Dual-Cycle Processes' Growth Factors (Study 2)

	Intercepts																			
	Commitment making				Identification with commitment				Exploration in breadth				Exploration in depth				Ruminative exploration			
Independent var.	b	*p*	SE	β	b	*p*	SE	β	b	*p*	SE	β	b	*p*	SE	β	b	*p*	SE	β
Self-event connect.	-.06	.592	.12	-.04	-.09	.365	.09	-.06	.15	.138	.10	.11	.05	.554	.09	.05	.18	.069	.10	.14
	Slopes																			
	Commitment making				Identification with commitment				Exploration in breadth				Exploration in depth				Ruminative exploration			
	b	*p*	SE	β	b	*p*	SE	β	b	*p*	SE	β	b	*p*	SE	β	b	*p*	SE	β
Self-event connect.	.00	.991	.06	.00	-.01	.899	.05	-.01	-.04	.349	.05	-.08	.01	.769	.05	.02	-.05	.357	.06	-.08

Table S7

Results of the Latent Growth Curve Model with Agency as Predictor of the Dual-Cycle Processes' Growth Factors (Study 2)

	Intercepts																			
	Commitment making				Identification with commitment				Exploration in breadth				Exploration in depth				Ruminative exploration			
Independent var.	b	*p*	SE	β	b	*p*	SE	β	b	*p*	SE	β	b	*p*	SE	β	b	*p*	SE	β
Agency	-.01	.930	.06	-.01	.01	.897	.05	.01	.01	.861	.05	.01	.08	.195	.06	.13	.01	.877	.05	.01
	Slopes																			
	Commitment making				Identification with commitment				Exploration in breadth				Exploration in depth				Ruminative exploration			
	b	*p*	SE	β	b	*p*	SE	β	b	*p*	SE	β	b	*p*	SE	β	b	*p*	SE	β
Agency	.07	.056	.04	.15	.06	.022	.03	.14	.06	.027	.03	.19	.06	.009	.02	.19	-.01	.772	.04	-.03

References

Byrne, B. M. (2013). *Structural equation modeling with Mplus: Basic concepts, applications, and programming*. New York, NY: Routledge.

Chen, F. F. (2007). Sensitivity of goodness of fit indexes to lack of measurement invariance. *Structural Equation Modeling: A Multidisciplinary Journal, 14*, 464-504. doi:10.1080/10705510701301834

Cheung, G. W., & Rensvold, R. B. (2002). Evaluating goodness-of-fit indexes for testing measurement invariance. *Structural Equation Modeling: A Multidisciplinary Journal, 9*, 233-255. doi:10.1207/S15328007SEM0902_5

Muthén, L. K., & Muthén, B. O. (1998-2015). *Mplus user's guide* (7th ed.). Los Angeles, CA: Author.

Satorra, A., & Bentler, P. M. (2001). A scaled difference chi-square test statistic for moment structure analysis. *Psychometrika, 66*, 507-514. doi:10.1007/BF02296192

Van de Schoot, R., Lugtig, P., & Hox, J. (2012). A checklist for testing measurement invariance. *European Journal of Developmental Psychology, 9*, 486-492. doi:10.1080/17405629.2012.686740

Widaman, K. F., Ferrer, E., & Conger, R. D. (2010). Factorial invariance within longitudinal structural equation models: Measuring the same construct across time. *Child Development Perspectives, 4*, 10-18. doi:10.1111/j.1750-8606.2009.00110.x
